# Neurofilament Light Chain (Nfl) Level in Serum as a Suitable In Vivo Biomaker for Axonal Damage in a Murine Viral Model of Multiple Sclerosis

**DOI:** 10.1111/jcmm.71304

**Published:** 2026-07-29

**Authors:** A. Segna, R. Wannemacher, K. Rohn, V. Sippel, M. A. Steiner, F. Lühder, A. Flügel, W. Baumgärtner, K. Hülskötter

**Affiliations:** ^1^ Department of Pathology University of Veterinary Medicine Hannover Hannover Germany; ^2^ Center for Systems Neuroscience Hannover Germany; ^3^ Department of Biometry, Epidemiology and Data Processing University of Veterinary Medicine Hannover Hannover Germany; ^4^ Idorsia Pharmaceuticals Ldt. Allschwill Switzerland; ^5^ Institute of Neuroimmunology and Multiple Sclerosis Research University Medical Center Göttingen Göttingen Germany

## Abstract

Neurofilament light chain (Nfl) is a protein of the cytoskeleton predominantly found in large calibre myelinated axons and a suitable in vivo biomarker for monitoring neurodegenerative processes. Axonal damage results in Nfl release into the cerebrospinal fluid and the blood stream. Axonal damage is a frequent finding in Theiler's murine encephalomyelitis virus (TMEV)‐infected SJL mice, a viral model for multiple sclerosis. Following the hypothesis that TMEV‐associated axonal damage is reflected by serum Nfl (sNfl) levels, histopathological findings were compared with sNfl levels at 7, 14, 42 and 147/85 days post infection (dpi) in TMEV‐susceptible SJL and ‐resistant C57BL/6 mice. Axonal damage was evaluated by immunohistochemistry for β‐amyloid precursor protein (βAPP) and synaptophysin (syn). Both markers were significantly increased at 42 and 85 dpi in the spinal cord of SJL mice, whereas no axonopathy was observed in the spinal cord of C57BL/6 mice. Similarly, significantly increased sNfl values were only observed in SJL mice at 42 and 85 dpi but not in C57BL/6 mice. Results show a positive correlation between histochemically detected axonal damage and sNfl in TMEV‐infected SJL mice. Thus, sNfl represents a potential marker for in vivo monitoring of axonal damage in this animal model.

## 1 Introduction

Neurofilaments are proteins of the cytoskeleton, which are specific for neurons and most abundantly expressed in large calibre myelinated axons, but also to some extent within synapses [1, 2]. In general, neurofilaments belong to the family of type IV intermediate filaments, which consist of four subunits, the neurofilament light chain (Nfl), the neurofilament medium chain (NfM), the neurofilament heavy chain (NfH), and α‐internexin [2]. Their main function is the maintenance of axon calibre, to ensure radial growth of axons during development and to facilitate the transmission of electrical impulses along axons [3]. In addition, a role in regulation of synaptic transmission, organelle trafficking, as well as synaptic turnover was described [4]. Nfl is the predominant neurofilament in axons and the most soluble subunit [5]. If the axonal membrane is disrupted, neurofilaments are released into the interstitial fluid [1, 2]. A constant release of Nfl from axons is also seen during the aging process due to physiologic degradation as well as concomitant to neuronal damage [6]. In contrast to age‐related axonal degeneration, disease‐ and trauma‐induced axonal damage triggers larger quantities of Nfl to be released via the extracellular space into the cerebrospinal fluid (CSF) and, after crossing the blood–brain‐barrier, into the blood stream [7]. The exact mechanism of neurofilament release into the blood stream is not yet fully understood [8]. Currently, a direct intramural periarterial drainage from the brain by perivascular pathways is proposed, similar to what has been described for amyloid‐β [9].

Serum Nfl (sNfl) analysis [10] is commercially in use as an early diagnostic and prognostic marker for patients with degenerative neurological diseases such as multiple sclerosis (MS), Alzheimer's disease, and amyotrophic lateral sclerosis (ALS), but also traumatic brain injury and perioperative neurocognitive disorders (PNDs) [5, 8, 10, 11, 12, 13, 14]. Although it does not replace MRI, which provides information related to the location of the CNS lesions in MS, sNfl measurement is a minimally invasive and cost‐effective tool for tracking neuroaxonal damage compared to MRI or cerebrospinal fluid analysis via lumbar puncture [15, 16].

The BeAn‐Strain of the Theiler's murine encephalomyelitis virus (TMEV) is a single‐stranded RNA virus with positive polarity and belongs to the family of *Picornaviridae*, genus *Cardiovirus*, species *Theilovirus* [17, 18] and serves as a valuable viral model to investigate the underlying mechanisms and pathogenesis of MS. The immune competence of the host, which is mediated via the presentation of immunogenic viral peptides by appropriate MHC‐I alleles, and the TMEV strain are essential factors for virus spread and persistence in the murine CNS after experimental intracerebral infection [19, 20, 21, 22]. The low virulent Theiler's original (TO)‐strains (TMEV‐BeAn or TMEV‐DA) result in an acute polio−/leukoencephalitis in susceptible mouse strains like SJL mice, followed by virus spread into the spinal cord and chronic demyelinating myelitis (TMEV‐induced demyelinating disease, TMEV‐IDD) mirroring MS lesions [19, 23, 24]. Disease development is accompanied by progressive loss of motor functions, starting in the hind limbs about 1 month after TMEV infection, myelin loss predominantly in the ventral white matter of the spinal cord and concomitant axonal degeneration [23, 25, 26, 27]. Resistant mouse strains, like C57BL/6 mice, develop an acute encephalitis with typical hippocampal lesions and can eliminate the virus from their CNS within 2 weeks after intracerebral infection and without spread into the spinal cord [19, 23]. Accordingly, they lack persistent infection and demyelinating spinal cord lesions [19, 21, 23, 28].

The axonal degeneration in TMEV‐IDD is characterized by axonal swelling, spheroid formation, and dilation of the myelin sheath in the spinal cord white matter [19, 29, 30] and accumulation of cytoskeletal components including beta amyloid precursor protein (βAPP) and/or components of synaptic vesicles like synaptophysin (syn) [29, 31]. To evaluate axonal damage in TMEV‐IDD, syn and βAPP are commonly used as immunohistochemical markers [32, 33, 34, 35, 36, 37].

Studies showed promising data, suggesting that sNfl might also be a suitable surrogate for axonal damage quantification within the spinal cord [9, 38, 39].

So far, axonal damage in TMEV‐IDD was mostly evaluated in the spinal cord using tissue samples from euthanized animals. Based on the studies in other neurodegenerative diseases, it is hypothesized that sNfl may represent a promising in vivo biomarker to monitor axonopathy, including TMEV‐IDD. The aim of this study was to correlate sNfl levels to immunohistochemical markers for axonal damage at various time points post TMEV‐infection in one TMEV‐susceptible (SJL) and one TMEV‐resistant (C57BL/6) mouse strain.

## 2 Materials and Methods

### 2.1 Mice and TMEV Infection

SJL/JCrHsd (SJL) mice were purchased from Envigo RMS GmbH, Rossdorf, Germany and from Charles River Gmbh, Sulzfeld, Germany. Mice with a C57BL/6 background (C57BL/6) were bred at the Institute for Neuroimmunology and Multiple Sclerosis Research of the University Medical Center Göttingen (UMG) Göttingen, Germany as well as at the Twincore GmbH, Hannover, Germany. During the experiment, mice were housed in individually ventilated cages (IVC) at the Department of Pathology of the University of Veterinary Medicine in Hannover with ad libitum feeding and water and a room temperature of 20°C–24°C with 50%–60% humidity and constant 12 h/12 h light and dark intervals. The SJL mice and C57BL/6 mice were infected intracerebrally at 5 weeks of age with 20 μL of TMEV‐BeAn‐solution with a concentration of 2.7 × 10^7^ plaque forming units per millilitre (PFU/mL) per animal [20]. The TMEV‐ solution was injected over a time period of 1 min into the right hemisphere of the brain as described before [40]. Prior to infection, mice were anaesthetised by intraperitoneal injection of 6 mg/kg ketamine and 0.45 mg/kg medetomidine. The control groups of SJL and C57BL/6 mice did not receive any treatment and were sacrificed accordingly. All experiments were performed in accordance with German law and approved by the Lower Saxony State Office for Consumer Protection and Food Safety as the responsible authority (Niedersächsisches Landesamt für Verbraucherschutz‐ und Lebensmittelsicherheit (LAVES), Oldenburg, Germany; AZ: 33.12‐42502‐04‐17; AZ: 33.8‐42502‐17/2418).

### 2.2 Necropsy and Tissue Sampling

SJL and C57BL/6 mice were euthanized at pre‐determined time points: at 7, 14, 42 and 147 days post infection (dpi). 147 dpi was the originally planned time point for the 4th necropsy date; however, due to elevated clinical scores in SJL mice, they already had to be euthanized at 85 dpi for humane reasons. Because experiments were not performed in parallel, the 4th necropsy date of the C57BL/6 mice remained 147 dpi. Euthanasia was performed via injection of a combination of 200 mg/kg ketamine and 1.0 mg/kg medetomidine. Blood samples were taken immediately after opening of the right atrium of the heart prior to perfusion. Blood serum was then collected after centrifugation at 4°C, at 3400 ***g*** over 5 min. Mice were post mortem perfused at a rate of 3.75 mL/min with phosphate buffered saline (PBS) [36].

Tissue samples were fixed in 10% buffered formalin for 24 h prior to paraffin embedding [36].

### 2.3 Histology and Immunohistochemistry

For immunohistochemistry (IHC), formalin‐fixed, paraffin‐embedded (FFPE) spinal cord parts were cut in 2 μm thick sections. Cervical, thoracic and lumbar spinal cord sections were evaluated separately as described before [36]. Sections were deparaffinized in Roticlear (C. Roth, cat. A538.3), isopropanol and 96% ethanol [36]. Immunohistochemistry was performed applying the avidin‐biotin‐peroxidase complex (ABC) method as described [41]. As pretreatment for immunohistochemistry, slides were placed in citrate buffer and microwaved for 24 min. βAPP‐ and syn‐positive axons were counted manually within the ventral medial and/or bilateral white matter of spinal cord sections of the cervical, thoracic, and lumbar spinal cord as described before [30, 32, 37]. The markers and the used dilutions for immunohistochemistry are summarized in Table 1. Tissue was derived from previous studies but has been evaluated specifically for the topic related matter of this analysis [36]. Evaluation of immunolabelled slides was performed on scans generated on a light microscope with camera (Olympus, DP72, Hamburg, Germany) or via light microscopy.

**TABLE 1:** The table shows antibodies, source, markers, dilutions and secondary antibodies used for the immunohistochemistry.

Antibody	Source and catalogue number	Marker for	Dilution	Blocking Serum	2nd Antibody (1:200)
Synaptophysin	Mouse anti‐human synaptophysin antibody, monoclonal, Dako, DAK‐SYNAP, M7315	Axonal damage	1:1000	Goat	Goat anti‐Mouse
Beta amyloid precursor protein (βAPP)	Mouse anti‐β‐ amyloid precursor protein A4 monoclonal antibody, monoclonal, Cat. Number: MAB348	Axonal damage	1:2000	Goat	Goat anti‐Mouse

### 2.4 Measurement of Neurofilament Light Chain (Nfl)

sNfl was quantified using a customized Meso Scale Discovery (Meso Scale Diagnostics, MSD, Rockville, Marland, USA) assay as previously described [42]. Plates (MSD, Rockville, MD) were coated overnight at 4°C with an anti‐Nfl capture antibody (Uman Diagnostics, Umea, Sweden). After blocking and washing, individual wells were incubated for 2 h at room temperature with 10 μL of the serum samples, followed by incubation with biotinylated anti‐Nfl detection antibody (500 ng/mL, 1 h) and SULFO‐TAG‐streptavidin (250 ng/mL, 1 h). Electrochemiluminescence was recorded after adding MSD read buffer on a Mesoscale Sector Imager 600 plate reader. Nfl serum concentrations were calculated from a bovine Nfl standard curve (10 pg/mL to 10 ng/mL).

### 2.5 Statistical Analysis

Statistical analysis was performed with SAS System (SAS Institute Inc.) and SPSS (IBM SPSS Chicago, IL, United States) for Windows. Graphs were created using GraphPad Prism (GraphPad Software). Assumptions of normal distribution of the response variables were tested using the Shapiro–Wilk test. Because the assumption of normal distribution was rejected, nonparametric methods were used. The Wilcoxon two‐sample test was used to compare two samples; for effects with more than two factor levels, the Kruskal–Wallis test was used with the post hoc Dwass, Steel, and Critchlow‐Fligner method for multiple pairwise comparison, while maintaining the experimental error rate. Associations between the outcome measures were calculated using Spearman's correlation. Statistical significance was accepted at exact *p*‐values less than 0.05.

## 3 Results

**FIGURE 1:**
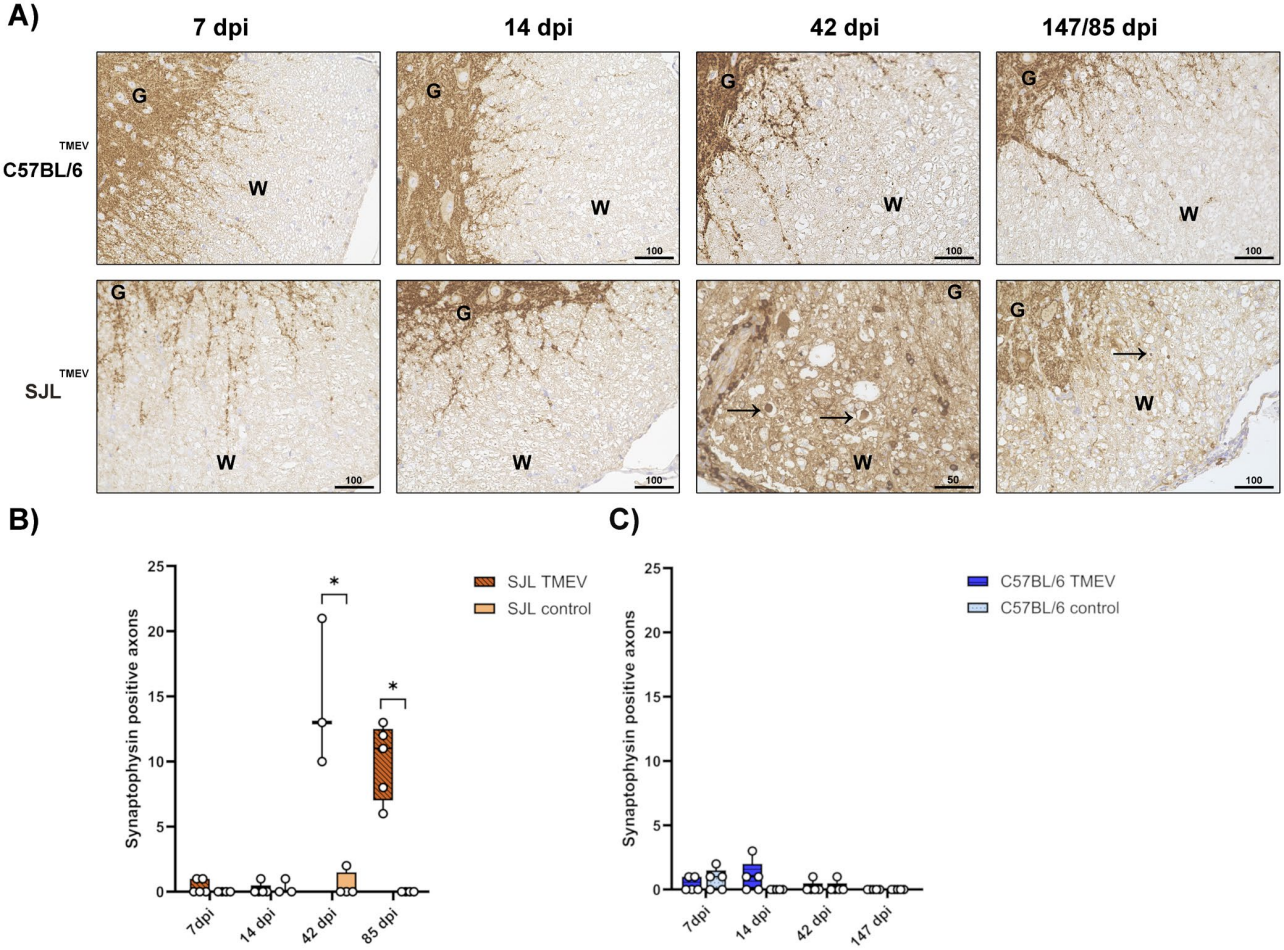
(A) Synaptophysin‐positive axons in the spinal cord of TMEV‐infected SJL and C57BL/6 mice. While TMEV‐infected C57BL/6 mice showed no increase in synaptophysin‐positive axons (7, 14, 42, 147 dpi), SJL mice displayed a significant increase in synaptophysin‐positive axons (arrows) indicating axonal damage at 42 dpi and 85 dpi. Immunohistochemistry (IHC)‐Synaptophysin, Bars = 100 μm; Numbers of synaptophysin (syn)‐positive axons in the spinal cord of Theiler's murine encephalomyelitis virus (TMEV) infected and non‐infected SJL and C57BL/6‐mice. (B) In the acute phase (7 and 14 dpi) TMEV‐infected SJL mice showed no significant increase in positive axon numbers compared to non‐infected SJL controls. At the chronic phase of TMEV‐IDD at 42 and 85 dpi, the number of syn‐positive axons rised markedly compared to non‐infected SJL controls (42 dpi: *p*‐value = 0.028; 85 dpi: *p*‐value = 0.011). *marks statistically significant differences with *p* < 0.05. (Kruskal–Wallis‐Test); (C) Throughout all time points, only few syn‐positive axons were observed with no significant differences between TMEV‐infected C57BL/6 mice and controls. *Marks statistically significant differences with *p* < 0.05 (Kruskal–Wallis‐Test); dpi, days post infection; G, grey matter; syn, synaptophysin; TMEV, Theilers murine encephalomyelitis virus; TMEV‐IDD, TMEV‐induced demyelinating disease; TMEV, TMEV‐infected; W, white matter.

**FIGURE 2:**
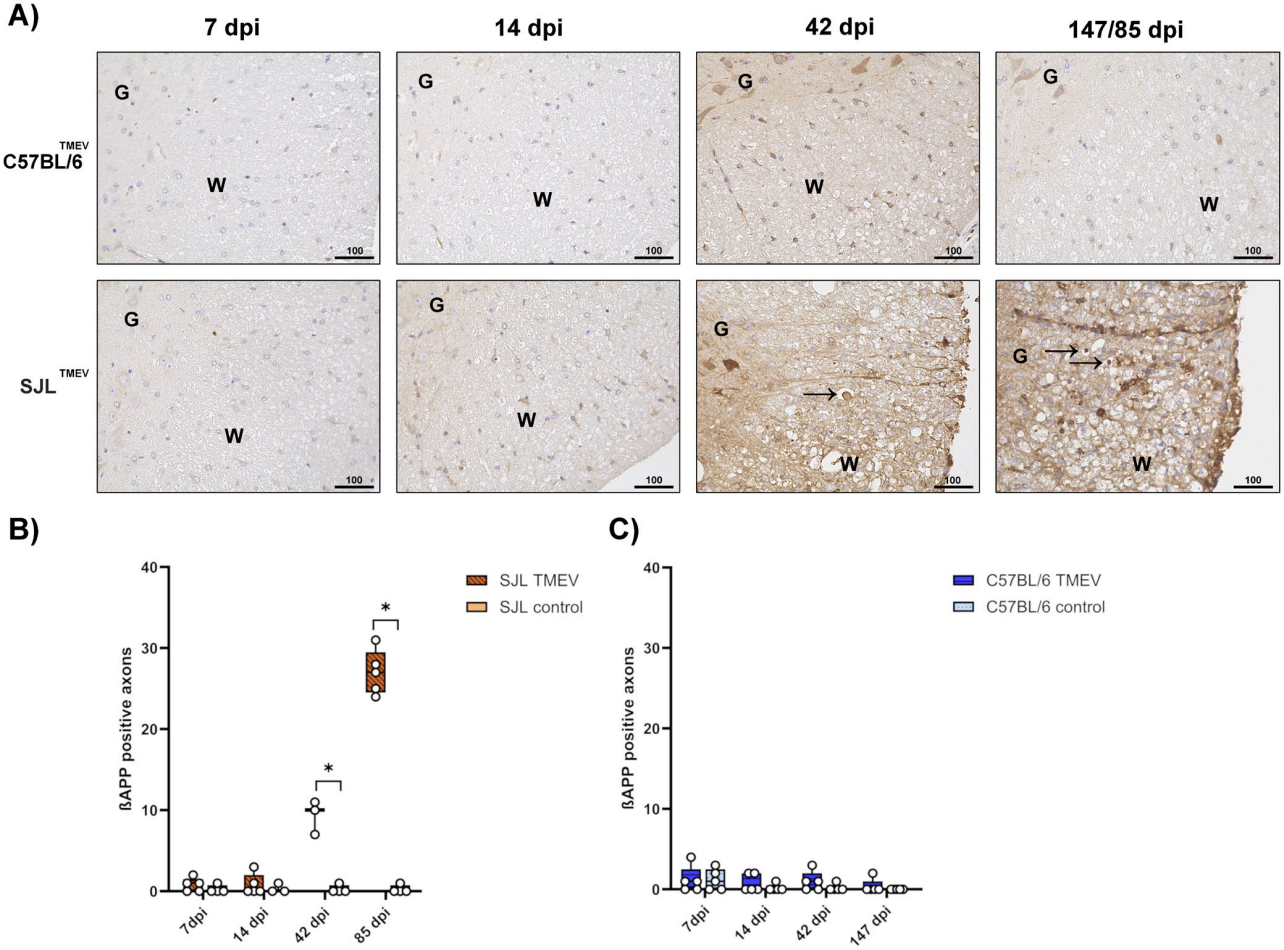
(A) βAPP‐positive axons in the spinal cord of TMEV‐infected SJL and C57BL/6 mice. There were substantial differences between immunohistochemical expression patterns of βAPP‐positive axons in TMEV‐infected SJL and C57BL mice. While TMEV‐infected C57BL/6 mice showed no increase of βAPP‐positive axons, SJL mice displayed a significant increase in βAPP‐positive axons (arrows) indicating axonal damage at 42 dpi and 85 dpi. Immunohistochemistry (IHC)‐ βAPP, Bars = 100 μm; (B) In the acute phase (7 and 14 dpi), TMEV‐infected SJL mice exhibited no significant elevation in positive axon numbers compared to non‐infected controls. At the chronic phase of TMEV‐IDD at 42 and 85 dpi, βAPP‐positive axons increased prominently compared to non‐infected SJL controls. (42 dpi: *p*‐value = 0,028; 85 dpi: *p*‐value = 0.013). *marks statistically significant differences with *p* < 0.05. (Kruskal–Wallis‐Test); (C) Throughout all time points, only few βAPP‐positive axons were observed with no significant differences between TMEV‐infected C57BL/6 mice and controls. *Marks statistically significant differences with *p* < 0.05 (Kruskal–Wallis‐Test); dpi, days post infection; G, grey matter; TMEV, Theilers murine encephalomyelitis virus; TMEV‐IDD, TMEV‐induced demyelinating disease; TMEV, TMEV‐infected; W, white matter; βAPP, β‐amyloid‐precursor‐protein.

In the first step, axonal damage was quantified using immunohistochemistry for syn and βAPP (Figures 1A and 2A).

The evaluation of syn‐positive axons in the spinal cord of TMEV‐infected and non‐infected SJL mice is shown in Figure 1B. In the acute phase of the disease at 7 and 14 dpi only few syn‐positive axons were observed in the spinal cord of TMEV‐infected SJL mice and non‐infected controls with no significant differences between the groups. In the chronic phase of the disease at 42 and 85 dpi, the numbers of syn‐positive axons in the spinal cord increased significantly in TMEV‐infected SJL mice compared to controls (42 dpi: *p*‐value = 0.028; 85 dpi: *p*‐value = 0.011).

In C57BL/6 mice (Figure 1C) only few syn‐positive axons were observed throughout the whole observation period at all time points with not significant differences between TMEV‐infected C57BL/6 mice and non‐infected controls.

Essentially the same results were obtained when evaluating βAPP‐positive axons in the spinal cord in TMEV‐infected and non‐infected SJL mice (Figure 2B). In the acute phase of the disease (7 and 14 dpi), only a small number of βAPP‐positive axons were observed in the spinal cords of both groups with no significant differences. In the chronic phase of the disease (42 and 85 dpi), the number of βAPP‐positive axons in the spinal cord is significantly increased in TMEV‐infected SJL mice compared to non‐infected controls (42 dpi: *p*‐value = 0.028; 85 dpi: *p*‐value = 0.013).

In C57BL/6 mice (Figure 2C), only a few βAPP‐positive axons were observed throughout all time points with no significant differences between TMEV‐infected C57BL/6 mice and non‐infected controls. Together with the histological analysis of syn, it can be concluded that in TMEV‐infected SJL mice massive axonal damage can be observed in the spinal cord in the chronic but not the acute phase after TMEV infection, whereas axonal damage cannot be detected in the spinal cord in TMEV‐infected C57BL/6 mice at any time point after TMEV infection.

**FIGURE 3:**
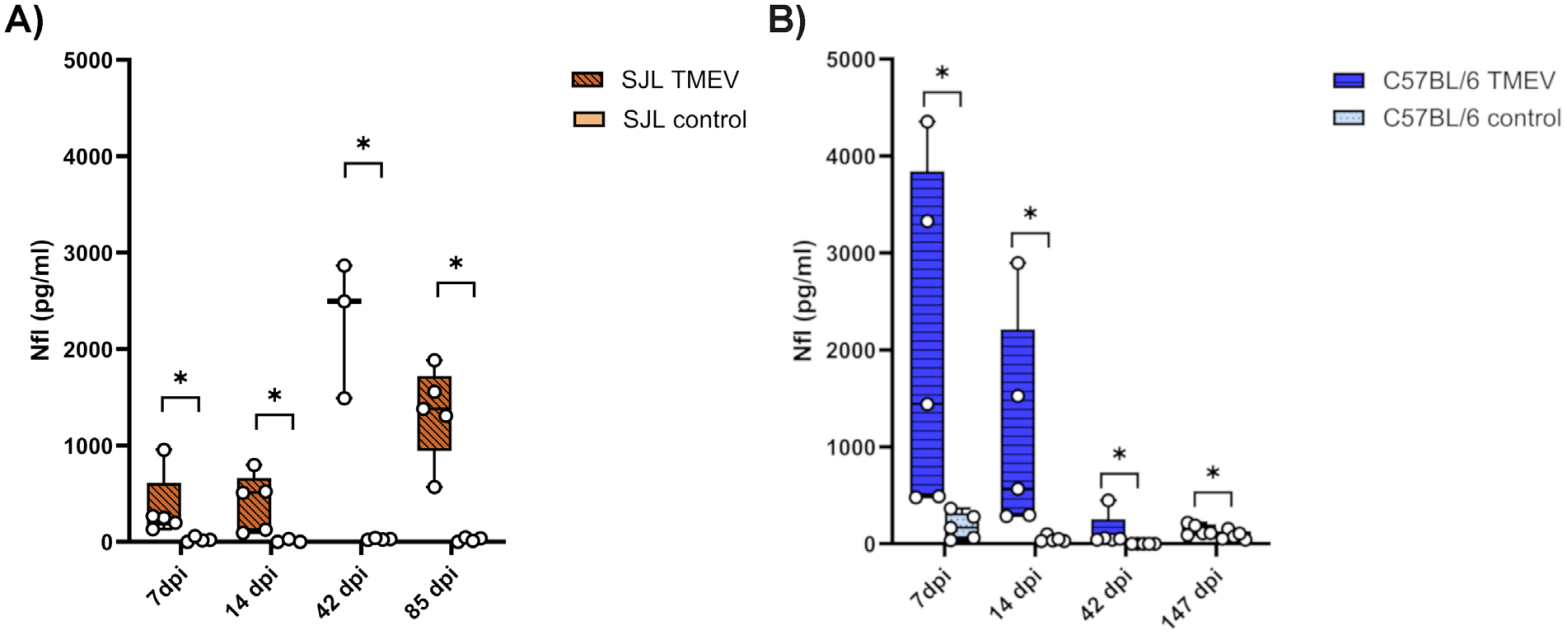
Level of neurofilament light (NfL) chain in Theiler's murine encephalomyelitis virus (TMEV)‐infected and non‐infected C57BL/6 and SJL‐mice. (A) In the acute phase (7 and 14 dpi), TMEV‐infected SJL mice exhibited already significantly elevated Nfl serum levels compared to non‐infected controls (7 dpi: *p*‐value = 0.014; 14 dpi: *p*‐value = 0.025). In the chronic phase of TMEV‐IDD at 42 and 85 dpi, neurofilament levels increased prominently compared to non‐infected SJL controls (42 dpi: *p*‐value = 0.034; 85 dpi: *p*‐value = 0.014). (B) In the acute phase (7 and 14 dpi), TMEV‐infected C57BL/6 mice exhibited significantly elevated Nfl serum levels compared to non‐infected controls (7 dpi: *p*‐value = 0.009; 14 dpi: *p*‐value = 0.009). At 42 and 147 dpi, neurofilament levels decreased compared to non‐infected controls but remained significant, although at 147 dpi the significance is only marginal (42 dpi: *p*‐value = 0.005; 147 dpi: *p*‐value = 0.046). *marks statistically significant differences with *p* < 0.05 (Kruskal–Wallis‐Test). dpi, days post infection; Nfl, neurofilament light chain; TMEV, Theilers murine encephalomyelitis virus; TMEV‐IDD, TMEV‐induced demyelinating disease.

In the next step, sNfl levels were measured in the same animal groups and analysed with respect to changes following TMEV infection and also in comparison to the immunohistochemical findings. The results for SJL and C57BL/6 mice are summarized in Figure 3.

TMEV‐infected SJL mice exhibit slightly but significantly elevated sNfl levels compared to non‐infected controls at 7 and 14 dpi (7 dpi: *p*‐value = 0.014; 14 dpi: *p*‐value = 0.025). During the chronic phase of TMEV‐IDD (42 and 85 dpi), sNfl values were markedly elevated in TMEV‐infected SJL mice compared to non‐infected controls (42 dpi: *p*‐value = 0.034; 85 dpi: *p*‐value = 0.014; Figure 3A).

TMEV‐infected C57BL/6 mice showed significantly elevated sNfl serum levels compared to non‐infected controls in the acute phase of the disease at 7 and 14 dpi (7 dpi: *p*‐value = 0.009; 14 dpi: *p*‐value = 0.009). However, sNfl levels decreased at 42 and 147 dpi compared to the values measured during the acute phase at day 7 and 14 dpi (42 dpi: *p*‐value = 0.005; 147 dpi: *p*‐value = 0.046). Non‐infected controls show no elevation in Nfl‐levels at all time points (Figure 3B).

Last, it was important to analyse a potential correlation between histological results and sNfl levels.

**FIGURE 4:**
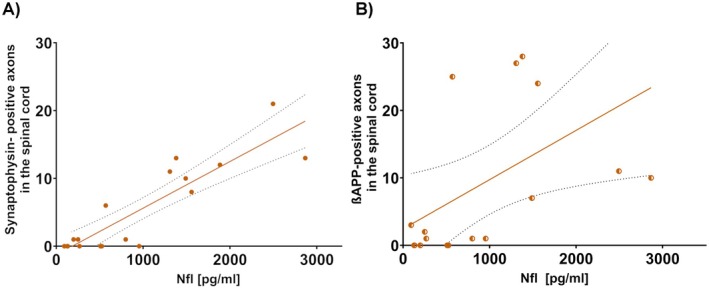
The scatterplots show the data points of TMEV‐infected SJL mice correlated to synaptohysin and β‐amyloid‐precursor protein. (A) In TMEV‐infected SJL mice, a Spearman correlation analysis revealed a significant positive trend between Nfl and synaptophysin, showing a positive correlation between immunohistological evaluated axonal damage and Nfl (*p*‐value = < 0.0001; *r* = 0.92092; *N* = 18). (B) In TMEV‐infected SJL mice, the Spearman correlation also showed a positive trend between Nfl and βAPP, thus showing a positive correlation between immunohistological evaluated axonal damage and Nfl (*p*‐value = 0.0017; *r* = 0.68657; *N* = 18). Nfl, neurofilament light chain, TMEV, Theilers murine encephalomyelitis virus, Spearman‐Correlation.

In TMEV‐infected SJL mice, a significant positive correlation between immunohistological evaluated axonal damage via syn and sNfl (*p*‐value = < 0.0001; *r* = 0.92092; *N* = 18; Figure 4A) as well as βAPP and sNfl was found (*p*‐value = 0.0017; *r* = 0.68657; *N* = 18; Figure 4B).

In TMEV‐infected C57BL/6 mice, results revealed a barely positive trend between sNfl and syn (*p*‐value = < 0.021; *r* = 0.4994; *N* = 21). No significant correlation was observed between sNfl and detected βAPP‐positive axons in the spinal cord of TMEV‐infected C57BL/6 mice (*p*‐value = 0.809; *r* = 0.0561; *N* = 21).

In the control groups of SJL and C57BL/6 mice, no positive correlation was revealed neither between sNfl and syn (C57BL/6 control: *p*‐value = 0.2161; *r* = 0.28926; *N* = 20; SJL control: *p*‐value = 0.9742; *r* = 0.00915; *N* = 15) nor between sNfl and βAPP (C57BL/6 control: *p*‐value = 0.2651; *r* = 0.26165; *N* = 20; SJL control: *p*‐value = < 0.0001; *r* = 1000; *N* = 15).

## 4 Discussion

The present study reveals a significant positive correlation of serum sNfl and the immunohistochemical markers for axonal damage syn and βAPP in TMEV‐infected SJL mice. At 42 dpi, in the chronic phase of TMEV infection, sNfl levels were significantly elevated in TMEV‐infected SJL mice. This increase was accompanied by a pronounced rise in βAPP‐ and syn‐positive axons observed at both 42 and 85 dpi by light microscopy. In contrast, the correlation of both immunohistochemical markers in the spinal cord and sNfl was not relevantly significant in non‐infected SJL controls, nor in TMEV‐infected or non‐infected C57BL/6 mice.

In summary, the results substantiate the hypothesis that sNfl represents a suitable in vivo biomarker to monitor axonopathies in TMEV‐IDD (42 and 85 dpi) and is most likely associated with the axonal damage in the spinal cord.

We assume that the slight decrease of syn at 85 dpi in TMEV‐infected SJL mice is related to the strong correlation between acute inflammatory axonal transport disturbances, microglial infiltration and syn‐positive axons, as previously described [32, 33, 34, 35, 37]. Usually, the syn signal decreases when inflammation is reduced and this is reflected by the strong correlation of our histopathological findings and the sNfl levels [34]. In SJL mice, the positive signal for syn was mostly located in demyelinated and microglia enriched areas [34]. βAPP was increasing until 85 dpi, which may be due to disturbances in different compartments of the axon [36]. The barely significant but positive correlation of sNfl and syn in TMEV‐infected C57BL/6 mice reflects a moderate linear association. Its biological significance remains questionable. The elevated sNfl levels in the acute phase are most likely the result of the neuronal loss and damage as well as inflammatory processes in the brain, predominantly in the hippocampus of TMEV‐infected SJL‐ and C57BL/6 mice as previously described [19, 32, 35, 36, 37]. In previous studies, it has been shown that at 7 and 14 dpi, the encephalitis in C57BL/6 and SJL mice is predominantly affecting the hippocampus due to an increased tropism for CA1 and CA2 pyramidal cell layers, leading to prominent inflammation, T cell infiltration and neuronal loss [19, 32, 35, 36, 37]. This acute phase usually resolves around 14 days post‐infection, which is reflected by the decrease in sNfl levels at 42 and 147 dpi in TMEV‐infected C57BL/6 mice [19, 35, 36, 37]. However, future studies should include the brain as target of TMEV to analyse the correlation between light microscopic changes and sNfl to substantiate this observation and conclusion. The sNfl levels of controls remained low at all time points; nevertheless, low amounts of sNfl were detected in the serum of most animals, which has been associated to aging and physiological synaptic turnover [6, 43]. Thus, it can be assumed that the large quantities of sNfl in SJL mice with TMEV‐IDD are released into the blood stream most likely due to the TMEV‐IDD ‐associated axonal damage [7, 9]. Our results align with a study in GM1 gangliosidosis mouse model, a lysosomal storage disorder characterized by impaired axonal transport, neuronal apoptosis, and myelin loss, where an accumulation of βAPP‐positive axons was detected in parallel with increased sNfL concentrations [44, 45].

Overall, this study also aligns with previous research in MS patients, where chronic white matter inflammation is associated with increased levels of sNfl in human patients [40, 46, 47, 48, 49]. Furthermore, it has been successfully correlated with disease modifying therapies as well as lesion development and progression in MS patients [15, 47, 50, 51]. The overall advantage of serum sNfl as a biomarker is strongly related to the fact that it can be simply quantified in a minimally invasive procedure, which can be repeated periodically and aids individualized therapy strategies [52].

Previous studies in various species indicate that the concentration in the blood stream is considerably lower than in the CSF [7, 48]. Nevertheless, many studies also showed a positive correlation between plasma or serum and the CSF in human patients [53, 54]. Interestingly, serum is predominantly chosen for neurodegenerative conditions like amyotrophic lateral sclerosis (ALS) or MS [7]. In contrast, plasma appears more frequently in dementia research [7]. Several publications have compared serum and plasma levels in human research, and it appears that both can be used interchangeably, as long as the values are standardized [7, 55].

Determination of sNfl, which represents a minimally invasive tool and provides a quantifiable parameter for analysis of axonal damage, could, depending on the study design and research question, reduce the number of animals required for experimental studies and, in addition, will allow an expansion of research objectives. However, it needs to be considered that sNfl does not provide any information on the location of axonal tissue damage [52]. Thus, depending on the point of interest, additional methods such as light microscopic analysis may still be required. The localization of spinal cord lesions in SJL mice affected by TMEV‐IDD has been extensively characterized, with the thoracic segment identified as the predominant site of pathology [19, 34, 56]. The available data significantly aids in the strategic planning and design of related studies [15, 40, 56]. Although blood samples in this study were obtained postmortem from the heart, the required volumes can be collected from sources such as the tail vein, depending on animal welfare guidelines and recommended sampling intervals.

It has been demonstrated that certain comorbidities affecting renal function or metabolic conditions, such as diabetes, can also influence experimental outcomes of sNfl levels, for example, reduced renal clearance has been reported in humans. This needs to be considered depending of the use of the animal model [57, 58].

## 5 Conclusions

In summary, this study provides a useful and easily accessible method for in vivo evaluation of axonal damage in TMEV‐IDD and allows sequential studies without the need to sacrifice animals at each time point. However, it needs to be considered that sNfl does not provide information on the location of tissue damage [52]. Yet, it seems to be an adequate in vivo marker to investigate axonal degeneration in this animal model and most likely other murine models of CNS‐related diseases.

## Author Contributions

**M. A. Steiner:** writing – review and editing, investigation. **V. Sippel:** investigation, writing – review and editing. **A. Segna:** conceptualization, writing – original draft, investigation, visualization, data curation, methodology. **A. Flügel:** conceptualization, investigation, writing – review and editing, methodology. **R. Wannemacher:** investigation, writing – review and editing, conceptualization, methodology. **K. Rohn:** data curation, formal analysis, writing – review and editing. **W. Baumgärtner:** supervision, conceptualization, validation, writing – review and editing, funding acquisition, investigation, methodology. **F. Lühder:** conceptualization, investigation, writing – review and editing, methodology. **K. Hülskötter:** supervision, conceptualization, validation, writing – review and editing, visualization, investigation, methodology, writing – original draft.

## Funding

The project was in parts financially supported by a scholarship from the Studienstiftung des deutschen Volkes for Anna Segna. This work was in part also supported by the Deutsche Forschungsgemeinschaft (DFG; German Research Foundation; 398066876/GRK 2485/1 and 2‐VIPER‐GRK). We acknowledge financial support by the Open Access Publication Fund of the University of Veterinary Medicine Hannover, Foundation.

## Ethics Statement

All experiments were performed in accordance with German law and approved by the Lower Saxony State Office for Consumer Protection and Food Safety as the responsible authority (Niedersächsisches Landesamt für Verbraucherschutz‐ und Lebensmittelsicherheit (LAVES), Oldenburg, Germany; AZ: 33.12‐42502‐04‐17; AZ: 33.8‐42502‐17/2418).

## Conflicts of Interest

The authors declare no conflicts of interest.

## Data Availability

The datasets used and/or analysed during the current study are available from the corresponding author on reasonable request.
